# Aiming at Optimal Physical Activity for Longevity (OPAL)

**DOI:** 10.1186/s40798-021-00360-4

**Published:** 2021-10-09

**Authors:** Martin Kopp, Martin Burtscher

**Affiliations:** Department of Sport Science, University of Innsbruck, Fürstenweg 185, 6020 Innsbruck, Austria

**Keywords:** Exercise, Fitness, Health, Age, Motivation

## Abstract

Although even small volumes (15–20 min) of daily physical activity (PA) are associated with health benefits, the optimal volume for healthy aging and longevity is substantially larger, amounting to about 100 min of daily moderate PA. The accomplishment of this objective likely requires the development of an appropriate PA lifestyle at an early age. Research initiatives are necessary addressing the motivational contribution of families, school surroundings and sport clubs, perhaps combined with role model effects and instructions for the specific implementation. Such approaches would need an enlarged agreement in readdressing this new aim followed by the launch of a new research strategy in order to develop specific offers for the respective age cohorts.

## Key Points

- Approaches to induce optimal rather than minimal amounts of physical activity are lacking.
- One possible approach to achieve optimal amounts of physical activity could be the foundation for high fitness levels and adherence to lifelong physical activity at an early age.

## Minimal Versus Optimal Physical Activity Recommendations

Regular physical activity (PA) represents the most important lifestyle component associated with cardiorespiratory fitness, healthy aging and longevity. In order to induce general health benefits, public health guidelines recommend, beside strength and balance exercises, at least 150–300 min of PA at moderate aerobic intensity or 75–150 min at vigorous intensity per week [1, 2]. Much more engagement in PA, however, is necessary to achieve maximal benefits on longevity [3]. For instance, a large prospective cohort study demonstrated maximal longevity gains at about 700 min of moderate or 350 min of vigorous PA per week [4]. The accomplishment of this objective is challenging in particular when considering that even minimal PA recommendations are difficult to achieve in the aging population [5]. Comprehensive programs have been developed to support older adults in reaching these minimal recommendations, e.g. through providing them with timely motivational messages and real-time feedback by smartphones and smartwatches [5]. In contrast, the evaluation of approaches intended to induce and maintain optimal rather than minimal PA recommendations is lacking but would be of utmost importance with regard to healthy aging and longevity. Such approaches likely require early adoption and maintenance of appropriate types and amounts of regular PA. For instance, lifelong aerobic exercise will not only impact on longevity but will also slow down the aging-related decline of general fitness. Remarkably higher cardiovascular and skeletal muscle metabolic fitness have been demonstrated among lifelong male and female exercisers in their 8th decade of life when compared to healthy non-exercisers [6]. The associated large physiological reserve above the frailty threshold supports not only individual independence but also participation in social life.

## Do Physical Activity Levels During Childhood and Adolescence Track into Adulthood?

From physiological as well as psychological perspectives, it makes sense to lay the foundation for high fitness levels and adherence to lifelong PA at an early age. A prerequisite, however, is that PA levels during childhood and adolescence track into adulthood. If this holds true, parents and physical education teachers could beneficially and importantly affect healthy aging and longevity. This has actually been demonstrated by several studies, indicating that PA lifestyle starts to develop in early childhood and that certain types of PA, e.g., running, and the consistency of participation during adolescence are associated with PA levels in adulthood [7–10]. Very recently, a randomized trial protocol has been presented examining motivational, regulatory, and habitual intervention approaches focusing on parent–child activities together [11]. The need of such interventions is underlined by study findings including monozygotic twins indicating beneficial long-term results by targeting childhood and adolescent environments like families and schools [12].

## Is Motivation the Key?

Motivation represents undoubtedly a critical factor for sustained exercise, with intrinsic motivation being especially important for long-term adherence [13]. Available data suggest that individuals with a high exercise adherence report positive exercise experiences including enjoyment in the past. Consequently, considerable importance is attached to the motivation by appropriate offers and continuation of PA and sports during childhood and youth [14]. Although motivational theories have considerably contributed to the increase of health enhancing PA, it will be a step forward to evaluate these theories in their efficacy on an optimal level of PA related to longevity (OPAL). Therefore, research initiatives addressing the motivational contribution of families, school surroundings and sport clubs, perhaps combined with role model effects and instructions for specific implementation, might be a promising path. The results will launch an educational gateway to sport and exercise, which may enable a lifelong flow between different motives to ensure OPAL, including health, social as well as performance related motives.

## Conclusions and Suggestions for Implementation

Although the transfer of recommended PA in childhood over the entire life span constitutes a target that is easy to understand and communicate, it requires joint and continuous efforts of decision-makers, including families, schools, sport clubs, healthcare providers, and policy-makers. First, easy access to child- and youth-oriented sports and PA possibilities, adapted to local conditions, constitutes a prerequisite. Further, a special focus must be set on the critical phase of the transition from school to working life, e.g., by specific sport offerings, including participation in sports groups, access to fitness studios, encouragement for participation in sporting events, etc. Moreover, the promotion of parent–child activities, appropriate sport and PA offerings for elderly individuals, but also disabled persons will help to prevent interruption of usual PA. These suggestions may not sound very revolutionary; however, convincing overall concepts and appropriate regimes for the control of their implementation are largely lacking.

Beside sport offerings, an abundance of evidence-based measures has been proved to effectively support PA behavior in various age and community groups. For instance, supporting the reduction of sitting and screen times will help to achieve higher PA levels, being of particular importance for adolescents [15]. Appropriate measures include a combination of motivational/volitional (e.g., health promotion by mass media), environmental (e.g., use of sit-stand or standing desks) and policy (e.g., curriculum change) strategies [16]. Promotion to increase every day physical activity like stair climbing was demonstrated to improve several health attributes, e.g., increased cardio-respiratory fitness, reduced risk of falling, and less perceived strain during daily life activities, particularly in the elderly [17]. Moreover, active commuting [18, 19] and gamification of PA constitute further promising applications to support PA behavior in healthy and diseased people as well [20]. Finally, societal changes such as city architecture and urban planning [21] or financial incentives [22] may effectively support the increase of PA and/or the improvement of adherence to PA recommendations.

The distinction between performance-related and health-related PA practice would have to be rethought and decision-makers would have to work systematically on an interaction between performance goals, social goals and health goals, backed with a metaplan for OPAL. Of course, this approach would need an enlarged agreement in readdressing this aim followed by the launch of a new research strategy in order to develop specific options for the respective age cohorts.

## Data Availability

Not applicable.

## Abbreviations

PA: Physical activity
OPAL: Optimal level of PA related to longevity
